# Collaborating for the Successful Retirement and End-of-Life Care of Non-Human Primates in Biomedical Research

**DOI:** 10.3390/vetsci11110560

**Published:** 2024-11-12

**Authors:** Amanda R. Maxwell, Eric K. Hutchinson, Jaclyn V. Allen, Melissa C. Painter, Lydia M. Hopper

**Affiliations:** 1 Department of Molecular and Comparative Pathobiology, Johns Hopkins School of Medicine, Baltimore, MD 21205, USA; 2 Research Animal Resources, Johns Hopkins University, Baltimore, MD 21205, USA; jallen67@jhmi.edu (J.V.A.); mpainte4@jhu.edu (M.C.P.); 3 Office of Laboratory Animal Medicine, University of Delaware, Newark, DE 19716, USA; hutchine@udel.edu

**Keywords:** *Macaca mulatta*, *Macaca nemestrina*, quality of life, veterinary care, behavioral management

## Abstract

Research facilities have established animal adoption programs for laboratory animals. However, adoption to private owners is not an option for non-human primates (NHPs), so their post-research life presents a unique challenge. Here, we describe a collaborative effort between laboratory animal veterinarians and behavioral management staff in retiring NHPs in place to ensure their quality of life after the completion of research projects. The success of our retirement program hinges on our collaborative efforts to manage clinical diseases such as arthritis, amyloidosis, and diarrhea; to surgically ex-plant previous research hardware; and to evaluate the animals’ behavior and temperament to ensure each individual is placed in a new group or living situation in which they are likely to be successful and to have good welfare. Implementation of a quality-of-life assessment tool for all retired animals allows all staff involved in the animals’ care to proactively and periodically assess the quality of life of these animals. The successful retirement of NHPs living in social groups is rewarding for all personnel and promotes a culture of caring. Research animal retirement is an important way for us to give back and honor the contributions these animals have made to advancing medicine.

## Simple Summary

Some scientific research necessitates the use of animal models, including monkeys. To honor the animals used in research, when possible, institutions seek opportunities to adopt out or retire their animals at the conclusion of studies. Primates, including macaque monkeys, are socially and psychologically complex animals and require specialized care from experts. Therefore, they cannot be adopted out to private owners and must only be retired to accredited facilities. However, retiring former research monkeys to sanctuaries can be costly to the institution and it may be stressful for the animals to move to an entirely new environment. Therefore, we developed a program to provide life-long care for retired macaques at our institution. Here, we describe how veterinarians, researchers, and behavioral managers collaborate to provide individualized care for primates in retirement. Our monkey retirement program allows for continuity of care for our monkeys, allows them to live with other macaques, and guarantees them individualized treatment plans for their clinical and behavioral needs. All retired animals are monitored closely to ensure they have continued quality of life into retirement.

## 1. Introduction

Non-human primates (NHPs) serve a very valuable role in biomedical research due to their anatomic and physiologic similarities to humans [1]. NHPs have contributed to various medical breakthroughs, including the development of the polio vaccine, insulin for diabetes treatment, and kidney dialysis [2]. There are many ongoing studies that will ultimately benefit both humans and animals, such as organ transplantation research and novel drug therapies [3]. Given the benefits we have gained from research with NHPs, it is our collective duty to care for them with the highest possible standards. This requires extensive care by veterinarians, animal care takers, and primate behavior experts due to the monkeys’ high cognitive function and complex social needs. Per the animal welfare regulations, institutions must have a psychological wellbeing plan to ensure the welfare of this highly sentient species [4]. Given their social and psychological complexity, there is also increased interest in evaluating the cumulative research experience of NHPs [5] and for identifying options for post-research care for animals not involved in terminal research protocols, including retirement to sanctuaries.

Unlike domestic or companion animals, when NHPs reach the end of a study, the options for their post-research care are limited as they are unable to be adopted out to private individuals. Retirement to sanctuaries is an option [6,7,8] but is often not financially feasible due to the cost of transport and lifetime care for these animals [8,9]. Therefore, the retirement of NHPs post research is not common practice [10]. In response to these limited options for retirement to external facilities, such as sanctuaries and zoos, and given unique challenges posed by NHPs used in biomedical research, we have created a retirement-in-place program in which eligible rhesus and pigtail macaques (*Macaca mulatta* and *M. nemestrina*) are allowed to live out their post-research lives being retired from invasive research activities [11]. We have leveraged our breeding colony to house retired animals in social settings there. However, to provide retirement examples for research facilities without breeding colonies that are home to large social groups of primates, we also highlight two examples of animals that we have retired in place at our research facility.

## 2. Materials and Methods

### 2.1. Retirement at Johns Hopkins University

At our institution, we define “retirement” broadly to encompass any animal that cannot be sold to external institutions or enrolled in future invasive research protocols. This includes animals that formerly served as participants in research, former breeders, and animals that we deemed were not clinically viable to act as research participants and were not valuable for breeding purposes. This also includes former research animals that were transferred to our facility from other institutions that we currently use as breeders due to their genetic value, but which we would never sell nor enroll in invasive research protocols in the future (in contrast with non-retiree breeders who are research-naïve and could become eligible for research enrollment). Retired animals may serve multiple purposes, such as acting as an “uncle/aunt” to juveniles at our breeding facility, as (vasectomized) “beta” males in breeding groups, or as a companion (“buddy”) monkey [11]. An “uncle” or “aunt” monkey refers to an older macaque that lives in a group of juvenile monkeys and acts as a role model, policing social conflicts and modeling appropriate social behavior to the younger monkeys. We have also housed retired “uncle” males who have been vasectomized in single-breeding-male breeding groups. This increases the social complexity of the breeding group and houses the retiree in a social setting, while ensuring paternity certainty. A “buddy” monkey is an animal that serves as a social partner for animals on experimental study, or those requiring long-term clinical care and monitoring, that otherwise would have been singly housed because no other potential partners were available at the time. Lastly, monkeys that do not originate from our breeding colony, and are of appropriate genetic stock, may act as breeders in one of our two active breeding colonies of pigtail and rhesus macaques. These animals undergo genetic testing through single-nucleotide polymorphism (SNP) testing and a physical examination and blood work by a veterinarian to ensure they are viable breeders. At our institution, we require macaques to have less than 6% Chinese origin in order to become breeders.

**Figure 1:**
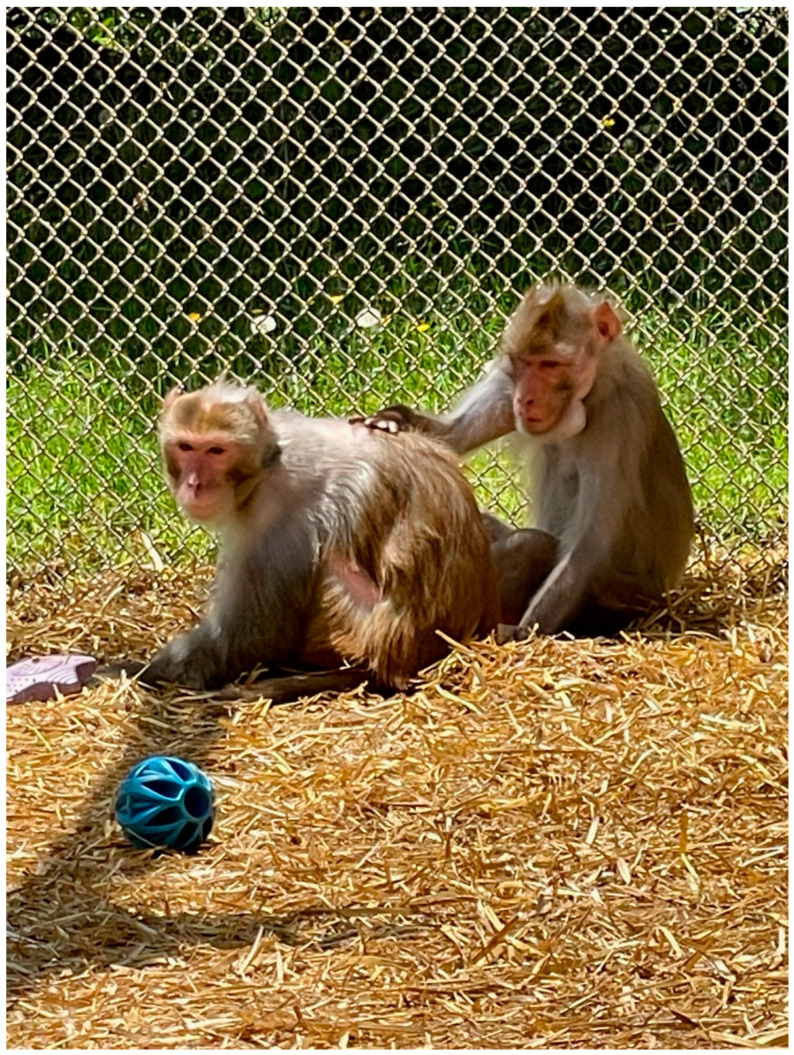
Two retired male rhesus macaques at the Johns Hopkins University breeding facility.

Over the years, we have retired both male and female pigtail and rhesus macaques. There are currently eleven male macaques enrolled in the Johns Hopkins retirement program, ranging in age from 4.5 to 27 years old, comprising eight rhesus macaques and three pigtail macaques. Six of the eleven macaques were previously on neuroscience studies; two macaques were failure-to-thrive animals that did not do well living in a large group setting at our breeding facility and were brought to our research facility to serve as companion monkeys; two were previously enrolled in a chronic pain treatment study; and one macaque was on a study for drug development (Figure 1). All eleven animals are socially housed, with nine living at our breeding facility in pairs or social groups in indoor/outdoor enclosures. The remaining two retirees (one pigtail, one rhesus) live at our research facility as buddy monkeys. Over time, the rhesus macaque buddy monkey has provided companionship to two adult females on research protocols, and the pigtail macaque has lived with two macaques requiring long-term clinical care. Additionally, there are currently 16 breeding rhesus macaques (10 males, 6 females) at our breeding facility that were sent to us from other facilities on the understanding that they would not be enrolled in research activities and who we also consider as retirees. 

The retired macaques may serve multiple roles throughout their retirement, balancing the needs of the colony with the behavioral needs and health status of the animal. For example, two retiree rhesus macaque males, one formerly in neuroscience and one in drug development, were originally used as breeders in our rhesus macaque breeding colony but now both serve as “uncles” living with groups of juvenile male rhesus that have been weaned from their natal groups. Another retiree, a vasectomized pigtail macaque, lived in a breeding group with a single breeding male and acted as a beta male, increasing social complexity, but currently lives with juvenile males as an “uncle”. Importantly, we prioritize social housing for all retired macaques and tailor their housing and social environments based on their individual behavioral and clinical needs. At the time of writing, all but one of the retiree macaques live in social groups and one is pair-housed with a long-term social partner. Additionally, in certain cases, our retirees offer social companionship to animals that might otherwise have been singly housed. 

Retirement in place has allowed us to ensure continuity of medical care for our animals and remove stressors that animals experience related to relocation, including transport stress, environmental change, and changes to personnel who provide care, as well as eliminate potential institutional concerns regarding messaging associated with relocating animals to an external facility [11]. Moreover, retirement in place is less financially costly as compared to retirement to an external facility. Specifically, we do not have to cover relocation costs associated with transferring animals to a new facility and, given our existing infrastructure, both in terms of personnel and housing, it is relatively cost-effective for us to retire animals in place at our facility. The lifetime care costs of all retiree animals are covered by our general operating funds. Moreover, when adding a few animals to existing groups, the cost of care for the entire colony is minimally increased and the benefits outweigh the minor financial costs associated with housing our retirees. Such benefits include the “uncles” providing guidance to juveniles to learn appropriate monkey behaviors or our buddy monkeys creating social support to animals on research studies.

### 2.2. Preparing for Retirement

The first step in our retirement program is identifying animals that would be good candidates for retirement. This is a collaborative effort between the veterinarians, the behavioral management team, and the principal investigators. Many criteria are used to identify animals for retirement including the clinical status of the animals, the behavior of the individual animal, and any potential ailments secondary to age or previous enrollment in research that may have long-term effects. Typically, clinically healthy animals will be enrolled in this program; however, animals with clinical ailments will be considered if the ailment does not impact the animal’s quality of life as determined by the veterinary and behavior teams. Animals with treatable illnesses, such as arthritis, will also be considered for retirement as long as the animal passes a veterinary assessment, which includes physical examination and blood work. The veterinarian will make a diagnosis and determine prognosis based on previous experience and expertise. 

The behavior team will conduct a behavioral assessment of the animal and will determine if the animal will likely have a good quality of life in retirement. The behavior team will evaluate the animal’s overall demeanor and any behavioral concerns, such as self-injury or previous history of social incompatibility, to determine both their suitability for retirement and what type of social and physical environment would best complement each individual animal (e.g., whether they should be pair- or group-housed, or what type or size of group they should be introduced to). The behavior team and veterinary team will communicate regarding any concerns and finalize an agreement on whether the animal is a retirement candidate and what specific, individualized care the animal may require if retired. 

The principal investigator (PI) is approached by the veterinary and behavior team about the possibility of research animal retirement, or, in some cases, the PI initiates the retirement processes for animals on their protocol. The experimental considerations are considered along with the experimental needs at the end of study. Animals that have experimental devices implanted may be retired with them in place, such as in the case of vascular access ports, or the devices may be removed, such as in the case of cranial implants. Johns Hopkins University (JHU) veterinarians have developed and previously presented a rotational skin-flap technique specifically designed to speed up recovery following the removal of cranial implant devices [12]. Given the success of the JHU animal retirement program, PIs are increasingly proactively reaching out to the veterinary and behavior teams to determine retirement plans for their animals and/or for securing buddy monkeys to offer companionship for animals on existing protocols. This highlights a cultural shift at our institution and a growing awareness of our retirement program, as well as the researchers’ strong support for it. Once an animal has been retired, all costs associated with the care of the animal are assumed by Research Animal Resources and are no longer a cost to the PI. 

### 2.3. Assessing Quality of Life in Retirement (QoLIR)

As for all animals under our care, we develop individualized health care plans for all retired macaques at our facility. One of the most common ailments observed in the retirees is arthritis, as they tend to be older animals. Arthritic animals are monitored using a mobility assessment tool, which comprises a number of simple criteria assessing the animal’s locomotion and attitude and scored on a scale from 1 to 5 (Appendix A). Such mobility scoring systems are used widely with geriatric primates (for a review, see [13]), as they are easy to implement and allow for real-time tracking of primates’ ease of movement and general wellbeing. 

In addition to monitoring specific disease conditions, like arthritis, that many of our geriatric retired animals have, we have also adapted the quality-of-life assessment tool described by Lambeth et al. (2013) to allow for holistic, long-term monitoring of the welfare of all retired macaques [14]. The intent of this process is to ensure active and continual monitoring of each retired macaque’s quality of life in retirement (QoLIR), not just to prompt end-of-life discussions. Such a process is especially important for macaques retired from research who may have participated in long-term or multiple protocols. Assessment of their cumulative life experience is key to understanding their welfare and suitability for retirement [5]. 

To initiate the assessment of each retiree’s quality of life in retirement (QoLIR), we form a team that comprises veterinarians, behavioral management staff, and care staff to discuss various quality-of-life indicators that are unique to each animal. For retired primates, it is important that this process is started at the point of retirement, not only when the animal presents with chronic health concerns, as successful retirement relies on the animal maintaining a good quality of life. As noted by Lambeth et al. (2013), the success of a quality-of-life assessment program hinges on input from veterinarians, behaviorists, and care staff [14]. Everyone at JHU who provides care for the retired animal is involved in the QoLIR discussions and is included in monitoring the animal’s wellbeing day to day. This ensures clear communication about the process as well as a detailed and thorough understanding of the parameters to be monitored and the factors that indicate good quality of life for each individual animal as well as parameters for end-of-life decisions. 

At our institution, the formation of the QoLIR team is led by a faculty veterinarian. The role of the QoLIR team is to discuss, monitor, and evaluate the animal’s quality of life. This process requires knowledge of the animal’s clinical and behavioral history (including reproductive success when applicable) to create QoLIR benchmarks and an evaluation framework for long-term monitoring. When the team first meets, they use a series of tools and metrics to assess the animal’s current quality of life, and they establish clinical and behavioral benchmarks for monitoring changes in quality of life over the course of their retirement. This process is not simply to evaluate end-of-life decisions, but also to ensure that once retired, each macaque maintains an overall positive quality of life. Once an animal is retired and placed on QoLIR watch, a number of factors can trigger re-evaluations, including changes in their clinical status, behavior, social relationships, and social housing status. Importantly, our QoLIR evaluations include metrics related to behavior and social relationships, in addition to clinical indicators, given the well-documented changes in primates’ social integration within their social groups with age [15,16].

During the initial QoLIR meeting, the faculty veterinarian explains the diagnosis of the animal of concern, including all clinical aspects of the case and any current signs or symptoms that may be identifiable to the team. These symptoms are recorded on the QoLIR Assessment Document (Appendix B). Additionally, Committee members who work closely with the animal provide input into the establishment of typical traits and behaviors for the animal. Programs such as the QoLIR Committees are intended to help prevent compassion fatigue experienced by all personnel, including care givers, veterinarians, technicians, and behavioral management staff, as they foster inter-team communication and ensure that people are kept informed about animal care decisions. 

We use two tools for the assessment of retired macaques adapted from those developed by Lambeth et al. (2013) for monitoring chimpanzee quality of life: (a) a behavioral ethogram and (b) a behavioral questionnaire (Appendix C and Appendix D) [14]. While clinical indicators can provide precise and objective measures, the use of behavioral questionnaires in assessing primate wellbeing have also been well validated across settings and species [17]. While we currently include standard clinical assessments in all our quality-of-life assessments, in the future we could include additional physiological measurements of long-term wellbeing, such as the level of glucocorticoids or measures of allostatic load. The behavioral recordings, together with these markers, could allow for a better evaluation of the animals’ wellbeing.

The macaque behavioral ethogram is a list of species-typical behaviors and abnormal behaviors (Appendix C). Committee members familiar with the animal’s daily activities rate and discuss whether and how frequently they observe the animal performing each behavior, on a scale that ranges from ‘never seen’ to ‘always seen.’ The behavioral questionnaire is a list of specific questions designed to stimulate discussion about the daily habits, responsiveness, unique characteristics, traits, and specific personality of the animal (Appendix D). The goal of this process is to determine a minimum of three behaviors or characteristics of the individual that would be noticeable if they changed and might confer a change in welfare. These are also documented on the QoLIR Assessment Document (Appendix B). 

At the initial QoLIR discussion meeting for each animal, the Committee determines set points or changes in the animal’s clinical and/or behavioral disposition that would trigger a discussion of changes in quality of life and, potentially, decisions related to euthanasia. These should be documented on the QoLIR Assessment Document. Importantly, the final decision to euthanize an animal rests with the faculty veterinarian.

Once the Committee has finalized the QoLIR Assessment Document, they establish a date for when the Committee will next reconvene. The frequency of Committee meetings varies from case to case and may change over time with the needs of each individual animal (e.g., meeting frequency might increase as an animal’s wellbeing changes more rapidly). Lastly, the Committee communicates to all staff members associated with the animal that a QoLIR Committee has been formed, the reason for forming the QoLIR Committee, and that a QoLIR Assessment Document has been created for the animal. A ‘QoLIR watch’ sign is placed on or near the animal’s enclosure that states (a) the animal’s ID; (b) diagnosis; (c) the faculty veterinarian who can be contacted for further information; and (d) instructions to contact the Committee representative if anything in this animal’s condition or behavior changes.

## 3. Results

We currently house 27 retired macaques at our facility, include those that are active breeders (n = 16) and those that are not breeders (n = 11). Once retired, animals are not available for any future invasive study or sale to external institutions as the ultimate goal of retirement is to extend their quality of life in retirement, not their use as an experimental model. We are continually seeking new opportunities to retire more animals in ways that benefit the retired individuals, the goals of the colony, and the JHU training and research program more broadly. This has required collaboration across many stakeholders and clear internal communication about the goals, benefits, and challenges of such a program. In addition to communicating the retirement plan to all involved stakeholders, our retired animals have “flags” in their electronic health records to indicate they are retired, and they are not to be used in another invasive experiment or sold for future research. As for all of the primates in our care, retired animals may have additional flags that denote any specialized care they require (e.g., specialized enrichment, particular social or behavioral needs), as we take an individualized approach to assessing and ensuring the welfare of these retired animals given their individual research histories and continued clinical and behavioral needs. These program goals are aligned with the Association of Primate Veterinarians’ Lifetime Use Guidelines [18].

The retired macaques have all lived at JHU for many years prior to retirement, and seven of the eleven non-breeding retired animals were born at our facility and so have lived their whole life under our care. A key benefit to retirement in place at our facility is that we can ensure continuity of care for all animals, and the JHU staff are intimately familiar with the macaques’ research history, clinical history, and behavioral needs [11]. For example, from a clinical perspective, staff are aware of historical sensitivities to sedation agents and how some animals may require alternative sedation agents or supportive medications to prevent sedation-associated nausea. Equally, from a behavioral perspective, our long-term knowledge of each animal’s social housing experience, rearing history, and behavioral needs allows us to prepare them for retirement and house them in the most suitable social environment for them to thrive.

As noted above, ensuring lifetime care for all retired macaques means that they represent an aging population, with an increasing number of the retirees being considered geriatric. We monitor those animals that show reduced activity or mobility fluency using our mobility scoring system. At our facility, any macaques requiring such monitoring (retirees or non-retirees) are scored daily by trained personnel. These scores are tracked over time, and an increasing score indicates that the mobility of the animal is worsening. Changes in scores will prompt a veterinary examination to rule out other causes of lameness, and medications will be added or adjusted as needed. Pharmacologic interventions include pain medications such as the GABA analog, gabapentin, and/or non-steroidal anti-inflammatories such as Galliprant^®^ (Elanco Animal Health, Inc., Greenfield, IN, USA) or carprofen. The behavior staff train the retirees to take medications in their social groups, ensuring their clinical needs are met while preserving their quality of life and social integration. Providing continued care to geriatric animals creates unique challenges that requires a holistic approach and also offers novel training scenarios for our laboratory animal post-doctoral fellows and potential opportunities for non-invasive, behavioral and cognitive studies of aging animals that further inform our care of such populations.

Retirement in place also maintains important human–animal bonds, which not only ensures continuity for the animals’ care but also enhances the experience for staff [19,20]. JHU promotes a culture of caring, and any new endeavors that enhance the wellbeing of staff members are greatly encouraged. Many members of the care staff, veterinary personnel, the behavioral management team, and lab members have reported the joy it brings them to see animals enter our retirement program, which in turn promotes job satisfaction and pride in their work.

## 4. Discussion

Through carefully selecting research animal retiree candidates, defining strategic roles for retirees, and promoting the teamwork and cooperation of dedicated care staff, veterinarians, and behavioral management staff, in-place retirement of non-human primates is an excellent and financially feasible option for animals retired from biomedical research [11]. The ability to retire these animals and provide a retirement with a good quality of life is an attainable goal we all should strive for to honor the animals that have contributed to the advancement of medicine [21]. Moreover, staff at our facility have reported to us the great satisfaction it gives them to see animals in retirement. This enhances their job satisfaction and helps to combat compassion fatigue. Given the very real concerns about compassion fatigue experienced by care staff and other personnel providing care for captive primates, including retired animals [22,23], we hope that involving multiple stakeholders in the QoLIR discussions helps foster inclusion and open dialog across parties so that all perspectives are heard and acknowledged. This process prioritizes and aims to foster both animal welfare and staff wellbeing.

## Data Availability

Assessment tools are provided in the Appendix A, Appendix B, Appendix C and Appendix D.

## Appendix B. Quality of Life in Retirement (QoLIR) Assessment Document

Animal ID:     Species:    DOB:

Housing location:    PI:        Protocol:

This document serves as a written record and guide as a basis for determining the ongoing assessment of this animal’s QoL and the timeliness of humane euthanasia for this animal. Today’s date:

**Physical/clinical signs to monitor:**

**Unique behavioral characteristics/preferences to monitor:**

Other Remarks:

**QoL Committee Members**

The QoL Committee, named below, has been assembled to determine behavioral parameters that may assist the faculty veterinarian in their consideration of humane euthanasia for this animal. The Committee comprises members who are intimately familiar with, or responsible for the care of, the animal.

**Faculty veterinarian** Name: ____________________ Signature:_____________________________

**LAM Postdoctoral Fellow** Name: ________________ Signature:_____________________________

**Veterinary Technician** Name: ___________________ Signature:_____________________________

**Behavioral Team Member** Name: ________________ Signature:____________________________

**Care Staff Member** Name: ______________________ Signature:____________________________

**Area Supervisor/Manager** Name: _________________ Signature:____________________________

## Appendix C. Macaque Behavioral Ethogram

**General NHP Behavioral Ethogram Discussion Tool**

*This is a tool to generate discussion about how frequently an animal engages in species-typical behaviors and other behavioral welfare indicators. Use this tool to generate behavioral indicators to be listed on the animal’s QoL Assessment Document.*

**Affiliates with others:** *May include grooming, huddling, embracing, or sitting in proximity to others*

Never	Sometimes	Frequently	Always

Has there been a recent change in this behavior? Yes, an increase Yes, a decrease No change

Comments:

__________________________________________________________________________

**Engages in Play:** *May include social play or solitary play, with or without objects*

Never	Sometimes	Frequently	Always

Has there been a recent change in this behavior? Yes, an increase Yes, a decrease No change

Comments:

__________________________________________________________________________

**Aggressive to others:** *May include threatening and chasing, as well as contact aggression*

Never	Sometimes	Frequently	Alway

Has there been a recent change in this behavior? Yes, an increase Yes, a decrease No change

Comments:

__________________________________________________________________________

**Submissive to others:** *May include SBTs, being displaced, fleeing or other species-typical behaviors*

Never	Sometimes	Frequently	Always

Has there been a recent change in this behavior? Yes, an increase Yes, a decrease No change

Comments:

__________________________________________________________________________

**Mates:** *Observed initiating or receiving mating behavior, may or may not include true breeding behavior*

Never	Sometimes	Frequently	Always

Has there been a recent change in this behavior? Yes, an increase Yes, a decrease No change

Comments:

__________________________________________________________________________

**Abnormal behaviors:** https://nprcresearch.org/research/page/BMC_-_Abnormal_Behavior_Ethogram (accessed on 4 November 2024).

Never	Sometimes	Frequently	Always

Has there been a recent change in this behavior? Yes, an increase Yes, a decrease No change

Comments:

__________________________________________________________________________

**Engages with enrichment:** *May include toys and food/foraging enrichment*

Never	Sometimes	Frequently	Always

Has there been a recent change in this behavior? Yes, an increase Yes, a decrease No change

Comments:

__________________________________________________________________________

**Engages with training/shows interest in familiar people**

Never	Sometimes	Frequently	Always

Has there been a recent change in this behavior? Yes, an increase Yes, a decrease No change

Comments:

__________________________________________________________________________

## Appendix D. Macaque Behavioral Questionnaire Discussion Tool

**Behavioral Questionnaire**

*The following list of questions aims to prompt discussion of consistent and predictable characteristics of the animal. Discussion of these questions will aid in the development of 3 or more unique behavioral characteristics to be listed on the animal’s QoL Assessment Document.*

Prompt: Consider each of these questions for the specific animal in question. Think about what behaviors are likely indicators of positive or poor welfare. Share if the animal has shown any marked changes in their behavior or personality.

1. What makes this animal unique? What behaviors do you think of when you think of this animal?
2. What words would you use to describe the animal’s personality/temperament?
3. How do other animals treat this animal?
4. If this animal takes medication or receives treatment, how reliably do they take their treatment?
5. How does this animal respond to potentially stressful situations like shifting or restraint?
6. How does this animal respond to daily husbandry tasks?
7. What is the activity level of this animal? Consider both rates and fluency of movement.
8. How well does this animal navigate social interactions? Especially during times of high arousal, such as during group fights or introductions
9. What are the typical eating patterns of this animal? Do they seek out food readily? Do they have particular preferences? Will they take treats from staff?
10. How does this animal use its enclosure? Do they have preferred or typical sleeping sites or postures? Do they avoid certain areas?
11. Does this individual display any behavioral indicators of pain or discomfort?
